# Comprehensive analysis of resistance-nodulation-cell division superfamily (RND) efflux pumps from *Serratia marcescens*, Db10

**DOI:** 10.1038/s41598-019-41237-7

**Published:** 2019-03-19

**Authors:** Shinsuke Toba, Yusuke Minato, Yuma Kondo, Kanami Hoshikawa, Shu Minagawa, Shiho Komaki, Takanori Kumagai, Yasuyuki Matoba, Daichi Morita, Wakano Ogawa, Naomasa Gotoh, Tomofusa Tsuchiya, Teruo Kuroda

**Affiliations:** 1 Department of Microbiology, Graduate School of Medicine, Dentistry and Pharmaceutical Sciences, Okayama University, Tsushima, Okayama 700-8530 Japan; 2 Department of Microbiology, Institute of Biomedical & Health Sciences, Hiroshima University, 1-2-3, Kasumi, Minami-ku, Hiroshima 734-8553 Japan; 3 Department of Microbiology and Infection Control Science, Kyoto Pharmaceutical University, 5, Misasaginakauchicho, Yamashina-ku, Kyoto 607-8414 Japan; 4 Present Address: Department of Microbiology and Immunology, University Minnesota Medical School, 689 23rd Avenue S.E., Minneapolis, MN 55455 USA; 5 Present Address: Department of Microbiology and Biochemistry, Daiichi University of Pharmacy, 22-1, Tamagawa-machi, Minami-ku, Fukuoka 815-8511 Japan

## Abstract

We investigated the role of the resistance-nodulation-cell division superfamily (RND) efflux system on intrinsic multidrug resistance in *Serratia marcescens*. We identified eight putative RND efflux system genes in the *S*. *marcescens* Db10 genome that included the previously characterized systems, *sdeXY*, *sdeAB*, and *sdeCDE*. Six out of the eight genes conferred multidrug resistance on KAM32, a drug hypersensitive strain of *Escherichia coli*. Five out of the eight genes conferred resistance to benzalkonium, suggesting the importance of RND efflux systems in biocide resistance in *S*. *marcescens*. The energy-dependent efflux activities of five of the pumps were examined using a rhodamine 6 G efflux assay. When expressed in the *tolC*-deficient strain of *E*. *coli*, KAM43, none of the genes conferred resistance on *E*. *coli*. When *hasF*, encoding the *S*. *marcescens* TolC ortholog, was expressed in KAM43, all of the genes conferred resistance on *E*. *coli*, suggesting that HasF is a major outer membrane protein that is used by all RND efflux systems in this organism. We constructed a *sdeXY* deletion mutant from a derivative strain of the clinically isolated multidrug-resistant *S*. *marcescens* strain and found that the *sdeXY* deletion mutant was sensitive to a broad spectrum of antimicrobial agents.

## Introduction

*Serratia marcescens*, a Gram-negative bacilli, is widely distributed in the environment. Although not initially regarded as a pathogen, *S*. *marcescens* is associated with occasional hospital-related outbreaks. The treatment of *S*. *marcescens* infections with antimicrobial agents is becoming more challenging because clinically isolated strains that exhibit elevated resistance against β-lactams, aminoglycosides, and fluoroquinolones have been reported^1,2^.

Resistance-nodulation-cell division superfamily (RND) efflux systems play a major role in multidrug resistance in Gram-negative bacteria^3–9^. RND-type efflux systems consist of three components: the inner membrane protein (IMP), periplasmic membrane fusion protein (MFP), and outer membrane protein (OMP). The electrochemical potential of H^+^ across the cell membrane appears to be the driving force for drug efflux associated with RND efflux systems. Three RND efflux systems in *S*. *marcescens*, SdeXY^10^, SdeAB^11^, and SdeCDE^11^, have been characterized to date. SdeXY was the first multidrug efflux system to be characterized from *S*. *marcescens*, and showed broad substrate specificity when expressed and characterized in *Escherichia coli*^10^. The gene expression of *sdeXY* was up-regulated in a tigecycline-resistant clinically isolated strain of *S*. *marcescens*^12^. The gene inactivation of *sdeXY* from the environmentally isolated *S*. *marcescens*-type strain, NCTC10211, increased susceptibilities to tigecycline, tetracycline, ciprofloxacin, and cefpirome. SdeAB also showed broad substrate specificity when expressed in *E*. *coli*^11^. Gene knockout analyses revealed that SdeAB conferred intrinsic multidrug resistance to fluoroquinolones, chloramphenicol, novobiocin, sodium dodecyl sulphate (SDS), and ethidium bromide in *S*. *marcescens*^13^. In a separate study, a *S*. *marcescens* cetylpyridinium chloride mutant showed the up-regulated expression of *sdeAB* and also became resistance to fluoroquinolones, tetracycline, chloramphenicol, and benzalkonium chloride^14^. In contrast to SdeXY and SdeAB, SdeCDE did not exhibit broad substrate specificity and only conferred novobiocin resistance to *S*. *marcescens*^15^.

In the present study, we aimed to identify uncharacterized *S*. *marcescens* RND efflux systems that have the potential to render *S*. *marcescens* with multidrug resistance. To achieve this, we examined putative *S*. *marcescens* RND efflux system genes from *S*. *marcescens* Db10 and characterized their substrate specificities in drug-hypersensitive *E*. *coli* strain KAM32^16^. We identified an additional three uncharacterized RND efflux systems with broad substrate specificities. A gene deletion analysis revealed that SdeXY conferred intrinsic multidrug resistance to *S*. *marcescens*.

## Results

### Cloning of putative RND-type efflux pumps from *S*. *marcescens*

**Figure 1:**
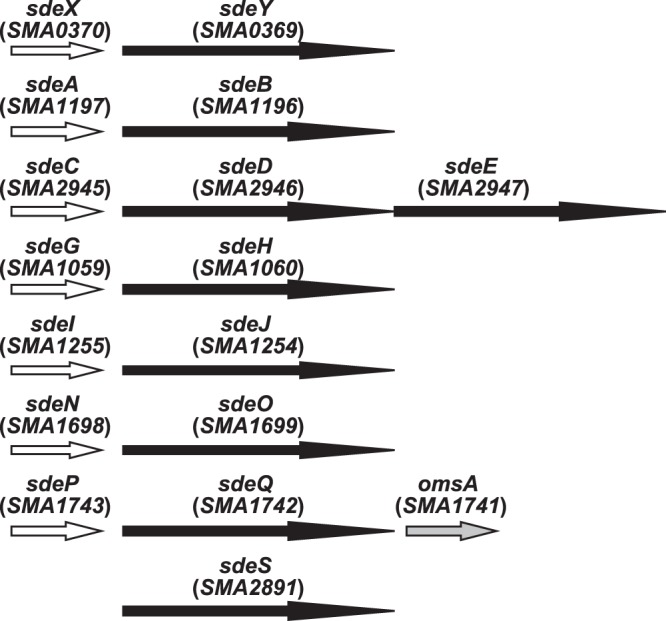
Putative *S*. *marcescens* RND efflux systems in Db10. White arrow; gene for the periplasmic membrane fusion protein, Black arrow; gene for the inner membrane protein, Gray arrow; gene for the outer membrane protein.

When we initiated this study, the *S*. *marcescens* Db11 genomic sequence database (http://www.sanger.ac.uk/resources/downloads/bacteria/serratia-marcescens.html) was the only publicly available resource for the genomic sequence of this bacterium. Using the *S*. *marcescens* Db11 genomic sequence, we searched for RND-type efflux systems in the *S*. *marcescens* Db11 genome and identified eight RND-type efflux systems (Fig. 1). These included three characterized *S*. *marcescens* RND efflux systems, SdeXY (SMA0370-0369)^10^, SdeAB (SMA1197-1196)^11^, and SdeCDE (SMA2945-2946-2947)^11^, and five putative RND-type efflux systems. We designated these putative RND efflux systems as shown in Fig. 1. The putative outer membrane protein (OMP) gene, *omsA*, was located adjacent to *sdePQ*. The other RND efflux systems did not contain the adjacent OMP gene. All of the RND efflux system genes contained the periplasmic membrane fusion protein (MFP) gene, except for *sdeS*. SdeCDE contained the two inner membrane protein (IMP) genes, *sdeD* and *sdeE*.

**Figure 2:**
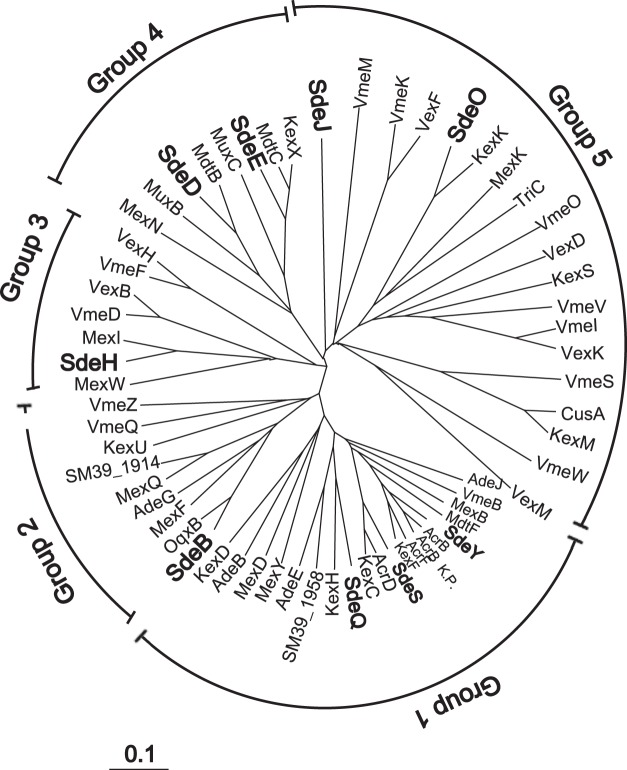
Unrooted phylogenetic tree of the inner membrane protein of RND efflux pumps. The phylogenetic tree was obtained using CLUSTALW (https://clustalw.ddbj.nig.ac.jp). *E*. *coli*: AcrB, AcrD, AcrF, MdtB, MdtC, MdtF, CusA^22,33,45,46^. *V*. *parahaemolyticus*: VmeB, VmeD, VmeF, VmeI, VmeK, VmeM, VmeO, VmeQ, VmeS, VmeW, VmeV, VmeZ^3–5^. *V*. *cholerae*: VexB, VexD, VexF, VexH, VexK, VexM^29,31^. *S*. *marcescens*: SdeB, SdeD, SdeE, SdeY, SdeH, SdeJ, SdeO, SdeQ, SdeS, SM39_1914, SM39_1958^10–15^. *A baumannii*: AdeB, AdeE, AdeG, AdeJ^17,18,20,26^. *P aeruginosa*: MexD, MexF, MexI, MexK, MexN, MexQ, MexW, MexY, MuxB, MuxC, TriC^21,24,27,28,30,32,34–36^. *K pneumoniae*: AcrB(K.P.), KexD, OqxB, KexC(KPN_RS15040), KexF(KPN_RS19875), KexH(KPN_RS21805), KexK(KPN_RS11560), KexM(KPN_RS25535), KexS(KPN_RS04245), KexU(KPN_RS03035), KexW(KPN_RS13595), KexX(KPN_RS13600)^19,23,47^.

We performed a dendrogram analysis of entire sequences of IMPs from *S*. *marcescens*, *E*. *coli*, *Vibrio parahaemolyticus*, *Vibrio cholerae*, *Klebsiella pneumoniae*, *Acinetobacter baumannii*, and *Pseudomonas aeruginosa*, and revealed that the IMPs from these organisms were divided into five groups (Fig. 2). In contrast to *E*. *coli* that does not have IMP in Group 2 or 3, each group contained at least one *S*. *marcescens* IMP, indicating that *S*. *marcescens* has a wide variety of RND efflux systems.

We cloned all *S*. *marcescens* RND efflux system genes from the *S*. *marcescens* Db10 strain, the parental strain of Db11, and expressed them in the drug-hypersensitive *E*. *coli* strain, KAM32, for further characterization.

### Substrate specificities of *S*. *marcescens* RND efflux systems

To assess the substrate specificity of each RND efflux system, we measured the MICs of various antimicrobial agents using strains expressing each RND efflux system gene(s) in *E*. *coli* KAM32 (Table 1).

**Table 1:** MICs of antimicrobial agents in *E*. *coli* KAM32 harboring each RND-type efflux pump.

Antimicrobial agent	MIC (µg/mL)											
	KAM32/pUC19	Group 1				Group 2	Group 3	Group 4			Group 5	
		KAM32/pURS2	KAM32/pURS8	KAM32/pURS82	KAM32/pURS9	KAM32/pURS3	KAM32/pURS5	KAM32/pURS4	KAM32/pURS44	KAM32/pURS45	KAM32/pURS6	KAM32/pURS7
	(control)	(*sdeXY*)	(*sdePQ*-*omsA*)	(*sdePQ*)	(*sdeS*)	(*sdeAB*)	(*sdeGH*)	(*sdeCDE*)	(*sdeCD*)	(*sdeCE*)	(*sdeIJ*)	(*sdeNO*)
Norfloxacin	0.016	0.06	0.13	0.06	0.016	0.06	0.13	0.03	0.03	0.03	0.016	0.016
Erythromycin	4	64	8	8	8	4	4	4	4	4	4	4
Chloramphenicol	1	8	1	2	1	8	1	1	1	1	1	1
Tetracycline	0.5	4	0.25	0.25	0.5	0.5	0.5	0.5	0.25	0.25	0.5	0.5
Benzalkonium Cl	4	32	8	4	4	16	32	4	4	4	32	4
Triclosan	0.5	2	0.25	0.13	0.5	4	8	0.5	N.D.	N.D.	0.5	0.5
Novobiocin	2	>128	16	16	8	4	4	8	2	2	2	2
SDS	128	>8200	>8200	256	>8200	256	1020	256	256	256	128	512
Deoxycholate	2050	32800	>32800	>32800	16400	2050	32800	2050	2050	2050	2050	2050
Ethidium bromide	4	>128	16	8	4	128	128	4	4	4	4	4
Rhodamine 6G	8	>128	>128	128	8	64	>128	8	8	8	>128	8

N.D.; not determined.

Many of the RND efflux systems in Group 1 play a major role in intrinsic multidrug resistance due to their broad substrate specificities^3,10,17–25^. Group 1 also contains the RND efflux system genes that are not in an operon with an MFP gene (e.g. *acrD* from *E*. *coli*). This type of RND efflux system generally exhibits narrow substrate specificities and SdeS only conferred resistance to erythromycin, novobiocin, SDS, and deoxycholate. Consistent with our previous findings^10^, the KAM32 strain expressing *sdeXY* conferred resistance to a broad spectrum of antimicrobial agents (Table 1). When *sdePQ* was expressed in the KAM32 strain with the adjacent OMP gene, *omsA*, multidrug resistance against several antimicrobial agents was conferred.

The RND efflux systems categorized into Group 2 have relatively broad substrate specificities and generally confer acquired resistance^4,11,13,14,26–28^. SdeB was categorized into this group and conferred multidrug resistance to *E*. *coli* KAM32; however, the substrate specificity of SdeAB was not as broad as those of SdePQ-OmsA and SdeXY (Table 1). The amino acid sequence of SdeB was similar to the *P*. *aeruginosa* RND efflux pump, MexF. Similar to MexEF^27^, fluoroquinolone and chloramphenicol were good substrates for SdeAB. Consistent with previous findings^11^, the KAM32 strain expressing *sdeAB* was resistant to the quaternary ammonium compound benzalkonium chloride.

The RND efflux systems in Group 3 have relatively broad substrate specificities and also confer acquired resistance^4,29–32^. Among *S*. *marcescens* RND IMPs, only SdeH was categorized into this group. The expression of *sdeGH* in *E*. *coli* KAM32 conferred multidrug resistance. The substrate specificity of SdeGH was broader than that of SdeAB, but was not similar to that of SdeXY or SdePQ-OmsA. Although SdeH showed amino acid sequence similarities to MexI and MexW, the substrate specificity of SdeGH was not similar to that of MexHI^32^ or MexVW^30^.

*sdeCDE* contained two IMP genes within its operon and SdeD and SdeE were both categorized into Group 4. As previously described^15^, novobiocin was the only substrate for SdeCDE (Table 1). To establish whether both of these IMP genes are required for this system, we constructed plasmids that expressed *sdeCD* or *sdeCE* and found that neither of these plasmids conferred novobiocin resistance in KAM32 (Table 1). This result indicated that SdeD and SdeE are both required for novobiocin resistance. This phenotype is similar to other RND efflux systems categorized into Group 4, such as MdtABCD from *E*. *coli*^33^ and MuxABC from *P*. *aeruginosa*^34^.

SdeJ and SdeO were categorized into Group 5. Although Group 5 contains many of the *Vibrio* RND efflux systems that show relatively wide substrate specificities^4,5,29,31^, the expression of *sdeIJ* conferred resistance to only benzalkonium and rhodamine 6 G, while *sdeNO* expression conferred resistance solely to SDS. TriC and MexK of *P*. *aeruginosa* were reported to contribute to the resistance of triclosan^35,36^. However, introduction of *sdeIJ* and *sdeNO* didn’t render triclosan resistance to host *E*. *coli*.

**Figure 3:**
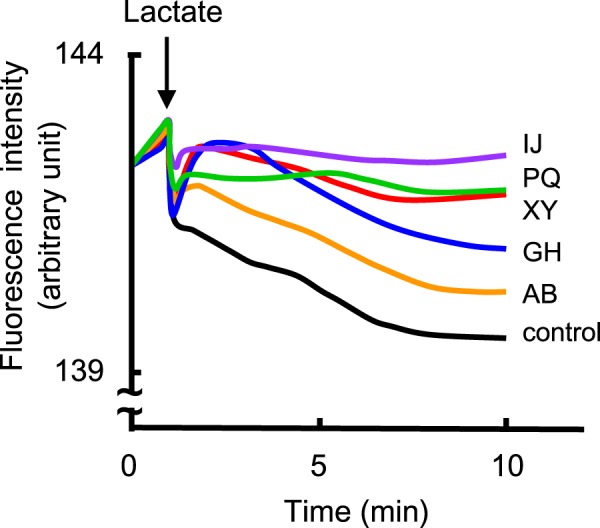
Rhodamine 6 G efflux assay. Energy-starved cells of *E*. *coli* KAM32 strains that express *S*. *marcescens* RND efflux systems were prepared as described in the Materials and Methods. Energy-starved cells were resuspended in PBS containing 5 mM MgSO_4_ and 1 µM rhodamine 6 G. At the time point indicated by the arrow, 20 mM potassium lactate (K-Lactate) was added to energize cells. Intracellular rhodamine 6 G levels were monitored continuously by measuring the fluorescence of rhodamine 6 G at excitation and emission wavelengths of 529 and 553 nm, respectively. IJ; *E*. *coli* KAM32/pURS6 (*sdeIJ*), PQ; *E*. *coli* KAM32/pURS8 (*sdePQ*-*omsA*), XY; *E*. *coli* KAM32/pURS2 (*sdeXY*), GH; *E*. *coli* KAM32/pURS5 (*sdeGH*), AB; *E*. *coli* KAM32/pURS3 (*sdeAB*), control; *E*. *coli* KAM32/pUC19.

We measured the efflux of rhodamine 6 G to evaluate the activity of each RND efflux system because rhodamine 6 G is a good substrate for most of the *S*. *marcescens* RND efflux systems (SdeXY, SdeAB, SdeGH, SdeIJ, and SdePQ-OmsA) (Fig. 3). All of these five efflux systems showed higher rhodamine 6 G efflux activities when lactate was provided as the energy source, indicating that rhodamine 6 G efflux systems are energy-dependent.

### Requirement of OMP

Except for *sdePQ*-*omsA*, the other eight *S*. *marcescens* RND efflux systems did not contain the adjacent OMP gene. Thus, these RND efflux systems must rely on *E*. *coli* OMP(s) when expressed in *E*. *coli*. TolC is a major OMP in *E*. *coli* and all of the *E*. *coli* RND efflux systems require TolC for their activities^37^. To clarify whether *S*. *marcescens* RND efflux systems utilize TolC when expressed in *E*. *coli*, we introduced *S*. *marcescens* RND efflux system genes into *E*.*coli* KAM43, a *tolC*-deficient strain, and tested antimicrobial susceptibilities by measuring MICs. Except for *sdePQ*-*omsA*, none of the *S*. *marcescens* RND efflux systems showed increases in MICs when expressed in KAM43, indicating that *S*. *marcescens* RND efflux systems require TolC for their activities when expressed in *E*. *coli* (Table 2).

**Table 2:** Effects of HasF on antimicrobial sensitivity.

Antimicrobial agent	MIC (µg/mL)										
			Group 1							Group 2	
	KAM43/pUC19	KAM43/pUC19/pSOS2	KAM43/pURS2	KAM43/pURS2/pSOS2	KAM43/p0URS82	KAM43/pURS82/pSOS2	KAM43/pURS8	KAM43/pURS9	KAM43/pURS9/pSOS2	KAM43/pURS3	KAM43/pURS3/pSOS2
	(control)		(*sdeXY*)	(*sdeXY*)	(*sdePQ*)	(*sdePQ*)	(*sdePQ*)	(*sdeS*)	(*sdeS*)	(*sdeAB*)	(*sdeAB*)
		(*hasF*)		(*hasF*)		(*hasF*)	(*omsA*)		(*hasF*)		(*hasF*)
Norfloxacin	0.016	0.032	0.016	0.064	0.016	0.016	0.032	0.016	0.016	0.016	0.032
Erythromycin	4	4	4	128	4	4	32	2	4	2	2
Benzalkonium	2	2	4	8	4	2	8	2	2	4	4
Novobiocin	0.25	4	0.5	32	0.5	4	32	0.5	2	0.5	2
SDS	32	64	32	>512	32	128	>512	32	128	32	128
Ethidium bromide	1	2	2	128	1	4	32	1	2	1	1
Rhodamine 6 G	4	8	4	>128	4	32	256	4	4	4	64
**Antimicrobial agent**	**MIC (µg/mL)**										
			**Group 3**		**Group 4**		**Group 5**				
	**KAM43/pUC19**	**KAM43/pUC19/pSOS2**	**KAM43/pURS5**	**KAM43/pUSR5/pSOS2**	**KAM43/pURS4**	**KAM43/pURS4/pSOS2**	**KAM43/pURS6**	**KAM43/pURS6/pSOS2**	**KAM43/pURS7**	**KAM43/pURS7/pSOS2**	
	**(control)**		**(** ***sdeGH*** **)**	**(** ***sdeGH*** **)**	**(** ***sdeCDE*** **)**	**(** ***sdeCDE*** **)**	**(** ***sdeIJ*** **)**	**(** ***sdeIJ*** **)**	**(** ***sdeNO*** **)**	**(** ***sdeNO*** **)**	
		**(** ***hasF*** **)**		**(** ***hasF*** **)**		**(** ***hasF*** **)**		**(** ***hasF*** **)**		**(** ***hasF*** **)**	
Norfloxacin	0.016	0.032	0.016	0.128	0.016	0.016	0.016	0.032	0.016	0.032	
Erythromycin	4	4	4	8	2	4	4	8	4	4	
Benzalkonium	2	2	4	16	2	4	4	4	2	4	
Novobiocin	0.25	4	0.5	4	0.5	8	0.5	4	0.5	4	
SDS	32	64	16	>512	32	64	32	256	32	256	
Ethidium bromide	1	2	2	64	2	4	1	16	1	32	
Rhodamine 6 G	4	8	4	>128	4	4	4	32	4	32	

We then investigated whether SdePQ utilizes TolC as an OMP. We subcloned *sdePQ* and expressed it in *E*. *coli* KAM 32 and KAM43. When expressed in *E*. *coli* KAM32, *sdePQ* increased MICs for several antimicrobial agents, similar to *E*. *coli* KAM32 expressing *sdePQ*-*omsA*, except for SDS (Table 1). However, when expressed in KAM43, *sdePQ* didn’t increase MIC for any agents. These results indicated that SdePQ utilizes TolC when OmsA is absent; however, this interaction may be weaker than that with OmsA (Table 2).

*S*. *marcescens* is known to possess the functional ortholog of TolC, HasF. Previous studies showed that SdeAB and SdeXY utilized HasF as their OMP component^12,13^. Since TolC may be utilized by all *S*. *marcescens* RND efflux systems, we hypothesized that the other *S*. *marcescens* RND efflux systems also utilize HasF as the OMP. To examine this, the *hasF* gene was cloned and expressed with *S*. *marcescens* RND efflux systems in the KAM43 strain. Consistent with previous findings, *sdeXY* and *sdeAB* both increased MICs when expressed with *hasF* in *E*. *coli* KAM43 (Table 2). When expressed with *hasF*, all of the *S*. *marcescens* RND efflux systems showed increased MICs, indicating that they also utilized HasF as the OMP. Compared Table 1 with Table 2, SdeAB-HasF and SdeNO-HasF showed higher MICs than SdeAB-TolC and SdeNO-TolC, whereas SdeIJ-HasF showed lower MICs than SdeIJ-TolC. These results indicated that compatibility between IMP and/or MFP and OMP is important.

Introduction of *sdeS* in KAM43 or KAM43/pSOS2 didn’t render the increase for any tested antimicrobial agents (Table 2), but in KAM32, increase of MICs for novobiocin, SDS, deoxycholate was observed (Table 1). Since no increase of MICs was observed in KAM33, an *acrA* disruptant of KAM32, SdeS would utilize AcrA as MFP in *E*. *coli* (Supplementary Table S1).

### The *sdeXY* deletion mutant of *S*. *marcescens* is susceptible to a broad range of antimicrobial agents

**Figure 4:**
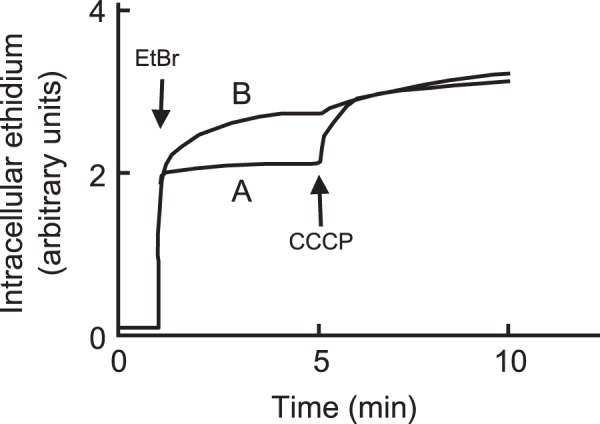
Ethidium efflux activity in *S*. *marcescens* cells. The cells of the *S*. *marcescens* KS24 strain (**A**) and its KS24∆*sdeXY* (**B**) were prepared as described in the Materials and Methods. Ethidium bromide was added to cell suspensions at a final concentration of 10 μM at the time point indicated by the first downward arrow. Intracellular ethidium levels were monitored continuously by measuring the fluorescence of ethidium at excitation and emission wavelengths of 500 and 580 nm, respectively. At the second downward arrow, CCCP was added to the suspensions at a final concentration of 100 µM. Assays were performed at 37 °C.

Since the present results indicated that SdeXY has the broadest substrate specificity among the characterized *S*. *marcescens* efflux pumps^10^, we constructed a *sdeXY* mutant strain from *S*. *marcescens*. We attempted to construct the deletion strain from Db10, but were unsuccessful for an unknown reason. Therefore, we used another strain KS24, a derivative of the clinically isolated strain of *S*. *marcescens* SM39^38^. The *sdeXY* deletion strain of KS24 became more sensitive to a broad spectrum of antimicrobial agents than the parental strain (Table 3). The *sdeXY* mutant strain also showed the decreased energy-dependent efflux of ethidium (Fig. 4). These results indicated that SdeXY is a major RND efflux pump that confers intrinsic resistance to *S*. *marcescens* against multiple antimicrobial agents.

**Table 3:** MICs of antimicrobial agents in *S*. *marcescens*.

Antimicrobial agent	MIC (µg/mL)			
	KS24	KS24Δ*sdeXY*	KS24Δ*sdeXY*/pSTV28	KS24Δ*sdeXY*/pSMXY
Cloxacillin	512	16	0.5	>512
Oxacillin	512	16	0.5	512
Erythromycin	256	2	2	256
Tetracycline	8	1	1	8
Chloramphenicol	8	1	N.D.	N.D.
Novobiocin	64	1	1	64
Norfloxacin	16	1	1	16
Ofloxacin	4	0.25	0.25	8
Ciprofloxacin	4	0.25	0.25	4
Benzalkonium chloride	32	2	2	32
Chlorhexidine gluconate	16	2	2	16
Triclosan	>1024	256	256	>1024
Acriflavine	128	16	16	256
Ethidium bromide	512	1	2	512
Hoechst33342	16	0.13	0.063	16
Rhodamine6G	1024	4	4	1024
TPP	4096	4	4	4096
Sodium cholate	>51200	3200	800	>51200
Sodium deoxycholate	3200	800	400	3200
SDS	51200	50	<25	51200

N.D.; not determined, TPP; tetraphenylphosphonium chloride.

## Discussion

Previous studies suggested that RND efflux systems play a major role in multidrug resistance in *S*. *marcescens*^10–14^. Since Gram-negative bacteria have been suggested to possess ‘multiple’ and ‘active’ RND efflux systems^3–9^, we hypothesized that *S*. *marcescens* has other ‘active’ RND efflux systems. To investigate this, we cloned all of the putative RND efflux systems from the *S*. *marcescens* Db10 strain, characterized them in *E*. *coli*, and further identified “active” *S*. *marcescens* RND efflux systems with broad substrate specificities.

A previous study suggested that SdeAB is the primary RND efflux system in *S*. *marcescens*, with the *sdeB* mutant becoming hypersensitive to multiple antimicrobial agents, similar to the *hasF* mutant^13^. However, the present study showed that SdeAB had narrower substrate specificities than SdeXY, SdePQ, and SdeGH. We also found that the *sdeXY* mutant became hypersensitive to a broad spectrum of antimicrobial agents. Furthermore, an independent study indicated that *sdeAB* was not expressed in the wild-type strain of *S*. *marcescens* and the expression of *sdeAB* was induced by the biocide, cetylpyridinium chloride^14^. Thus, SdeAB may play a primary role in multidrug resistance only in specific strains of *S*. *marcescens* and/or a strain that is exposed to a specific biocide.

We identified two previously uncharacterized *S*. *marcescens* RND efflux systems, SdePQ-OmsA and SdeGH, which exhibit broad substrate specificities. Our reverse transcription-PCR analyses on the *S*. *marcescens* Db10 and KS24 strains under normal growth conditions revealed that *sdeQ* and *sdeH* gene expression was not detected, while *sdeY* gene expression was observed in both strains (data not shown). When some mutations were occurred which caused the expression of SdePQ-OmsA and SdeGH systems, these pumps would contribute to the acquired resistance in *S*. *marcescens*.

All of the *S*. *marcescens* RND efflux systems utilized TolC and HasF when expressed in *E*. *coli*. However, some RND pumps when expressed with TolC of *E*. *coli* or HasF of *S*. *marcescens* showed different substrate specificities. SdeNO expressed with HasF in KAM43 showed higher MICs than that expressed in KAM32 with TolC, whereas SdeIJ and SdeS showed lower MICs. These results suggest that compatibility between outer membrane proteins and other components in RND pumps is important for its efflux activity. As our group reported previously, only VmeAB in *V*. *parahaemolyticus* showed high MICs expressed with TolC of *E*. *coli*, while other RND pumps in *V*. *parahaemolyticus* had markedly higher MICs when expressed with VpoC, an orthologue of TolC^4^. This result may be important for understanding the interaction between outer membrane proteins and other components.

In summary, the present study revealed that *S*. *marcescens* has multiple RND efflux systems that have the potential to confer multidrug resistance. Among these systems, SdeXY plays a major role in intrinsic multidrug resistance in *S*. *marcescens*. SdePQ-OmsA and SdeGH showed broad substrate specificities similar to SdeXY; however, these systems appear to be inducible and do not play major roles in the intrinsic multidrug resistance of *S*. *marcescens*.

## Materials and Methods

### Bacterial strains and growth conditions

The bacterial strains and plasmids used in the present study are listed in Supplementary Table S2. Unless otherwise noted, bacterial cells were grown in Luria (L) medium (1% polypeptone, 0.5% yeast extract, 0.5% NaCl, pH 7) at 37 °C under aerobic conditions. Antibiotics were supplemented when required as follows: ampicillin, 100 µg/ml; chloramphenicol, 20 µg/ml.

### Phylogenetic tree of IMPs

Entire sequences of inner membrane protein were obtained from several database. The phylogenetic tree was obtained using CLUSTALW (https://clustalw.ddbj.nig.ac.jp).

### Cloning, sequencing, and gene manipulation

We identified putative *S*. *marcescens* RND efflux system genes using the *S*. *marcescens* Db11 genomic sequence database (http://www.sanger.ac.uk/resources/downloads/bacteria/serratia-marcescens.html). DNA fragments, which contained the open reading frames (ORFs) of *S*. *marcescens* RND efflux system genes or *S*. *marcescens* OMP, *hasF* (*SMA3509*), were amplified by PCR using the chromosomal DNA of *S*. *marcescens* Db10, the parent strain of Db11^39^, as a template. Primers used for cloning are listed in Supplementary Table S3. Each primer included a restriction enzyme recognition site (underlined). The PCR products obtained were digested with the indicated restriction enzymes, gel-purified, and then ligated into the same restriction enzyme sites of the vector pUC18, pUC19, or pSTV28 (for *hasF*), which were located downstream of the *lac* promoter of the vector plasmid. Since PCR products did not include the promoter region of the genes, gene expression was controlled by the *lac* promoter.

The cloning of *sdeCDE* (SMA2945-2946-2947) was performed in two steps. The 5′ half fragment was amplified with two primers (SMA2945-2947F fw EcoRI and SMA2945-2947F re BamHI), and the 3′ half was then amplified with two primers (SMA2945-2947B fw EcoRI and SMA2945-2947B re BamHI). After digestion with *Eco*RI and *Bam*HI, each fragment was individually inserted into pUC18. The resultant plasmids were designated as pURS4F and pURS4B. After the digestion of pURS4B with *Mlu*I and *Bam*HI, the fragment was inserted into pURS4F at the same sites. It was named pURS4. The plasmid pURS44 carrying *sdeC* and *sdeD*, but not *sdeE* was also constructed. The plasmid pURS4 carrying *sdeC-sdeE* was digested with *Nco*I and self-ligated. Similarly, the plasmid pURS45 carrying *sdeC* and *sdeE*, but not *sdeD* was constructed. The plasmid pURS4 was digested with *Tth*111I, blunted, and self-ligated.

To evaluate the function of OmsA, the gene of which is located downstream of *sdePQ* (SM1743-1742), two types of plasmids were constructed. The fragment including *sdePQ-omsA* was amplified with PCR using SMA1743-1741 fw XbaI and SMA1743-1741 re EcoRI. After digestion with *Xba*I and *Eco*RI, the fragment was inserted at the same site in pUC19, and the resultant plasmid was named pURS8. To construct pURS82 carrying incomplete *omsA*, pURS8 was digested with *Hpa*I and self-ligated.

Since *S*. *marcescens* showed higher β-lactam resistance, we were unable to utilize pURS2 carrying *sdeXY*. To complement *sdeXY*, we constructed another plasmid pSMXY. PCR was initially performed with two primers SMA0370-0369 fw EcoRI and SMA0370-0369 re XbaI using the Db10 genome as a template. PCR was then performed with two different primers, SMA0370-0369 fw EcoRI and SMA0370-0369 re BglII, to create the *Bgl*II site instead of the *Xba*I site. The PCR products obtained were digested with *Eco*RI and *Bgl*II, gel-purified, and then ligated into the *Eco*RI-*Bam*HI sites of the vector pSTV28.

### Minimum inhibitory concentrations (MICs)

The MICs of various antimicrobial agents were assessed in Muller–Hinton broth (Difco) using the standard two-fold dilution method as previously described^40^.

### Construction of the *sdeXY* deletion strain

*S*. *marcescens* KS24, a plasmid pSMC2-cured derivative of *S*. *marcescens* KS3^41^ was used to construct a *sdeXY* deletion strain. The *sdeXY* deletion strain of *S*. *marcescens* KS24 was constructed by homologous recombination using the lambda Red recombinase system^42^. Two-step PCR of the gentamicin cassette flanked by long (1000 nt) homologous extensions of the target gene were essentially performed as previously described^43^ using pBRFRTGM and the genomic DNA of *S*. *marcescens* KS24 as the template and the primers listed in Supplementary Table S3. The resulting PCR product was separated on an agarose gel and purified using GENECLEAN II KIT (MP Biomedicals Inc.). *sdeXY* mutant strains were generated by electroporation of the purified PCR product into *S*. *marcescens* KS24/pKD46 as described previously^42^. The deletion of s*deXY* in the mutant strain was verified by PCR.

### Measurements of rhodamine 6 G and ethidium efflux activities

The efflux of rhodamine 6 G and ethidium was evaluated as previously described^9,40^. In the rhodamine6G efflux assay, *E*. *coli* KAM32 strains were grown in L media until O.D_650_ = 0.7. *E*. *coli* cells were harvested by centrifugation, washed twice using Potassium Phosphate Buffer (PPB) containing 5 mM MgSO_4_, and resuspended in the same buffer that contained 1 µM rhodamine 6 G and 40 µM carbonylcyanide-*m*-chlorophenylhydrazone (CCCP). The cell suspension was incubated at 37 °C for one hour to de-energize cells, washed twice using the same buffer that did not contain CCCP, and then resuspended in the same buffer. The resultant cell suspension was incubated on ice for two hours and used in the efflux assay.

In the ethidium efflux assay, the *S*. *marcescens* KS24 and *S*. *marcescens* KS24Δ*sdeXY* strains were grown in L media until O.D_650_ = 0.7. *S*. *marcescens* cells were harvested by centrifugation, washed twice using modified Tanaka Buffer^44^, and resuspended in the same assay. The resultant cell suspension was incubated at 37 °C in the presence of 20 mM lactate-tetramethylammonium hydroxide (pH 7.0) for 5 min and used in the efflux assay.

## Supplementary information

Supplementary table
