# Supplementary material for: Comprehensive analysis of resistance-nodulation-cell division superfamily (RND) efflux pumps from Serratia marcescens, Db10
Source: Sci Rep. 2019 Mar 19;9:4854. doi: 10.1038/s41598-019-41237-7 (PMC6425002; doi:10.1038/s41598-019-41237-7)
Supplement: Supplementary file 1 — Supplementary table [file 41598_2019_41237_MOESM1_ESM.docx]

**Comprehensive analysis of resistance-nodulation-cell division superfamily (RND) efflux pumps from *Serratia marcescens*, Db10**

Shinsuke Toba, Yusuke Minato, Yuma Kondo, Kanami Hoshikawa, Shu Minagawa,

Shiho Komaki, Takanori Kumagai, Yasuyuki Matoba, Daichi Morita, Wakano Ogawa,

Naomasa Gotoh, Tomofusa Tsuchiya, and Teruo Kuroda

**Supplementary Table S1**

**MICs of antimicrobial agents in *E. coli* KAM33 harboring SdeS**

MIC (µg/mL)

KAM33/pUC19 KAM33/pURS9

Antimicrobial agent (control) (*sdeS*)

Erythromycin 4 4

Tetracycline 0.25 0.25

Benzalkonium Cl 4 4

Triclosan 0.5 0.5

Novobiocin 2 2

SDS 128 128

Deoxycholate 1024 1024

Ethidium bromide 4 4

Rhodamine 6G 8 8

KAM33 : TG1, ∆*acrAB*, ∆*ydhE* ^3,4^

**Supplementary Table S2**

**Bacterial strains and plasmids used in the present study**

Strain/plasmid Description Sources

*E. coli*

KAM32 TG1, ∆*acrB*, ∆*ydhE* ^16^

KAM43 TG1, ∆*acrAB*, ∆*ydhE*, ∆*tolC* ^3,4^

*S. marcescens*

Db10 ^39^

KS3 pSMC1-cured derivative of SM39 ^41^

KS24 pSMC2-cured derivative of KS3 This study

KS24ΔsdeXY *sdeXY* deletion strain from KS24 This study

Plasmid

pUC18 Cloning vector, amp^r^

pUC19 Cloning vector, amp^r^

pSTV28 Cloning vector, Cm^r^

pBRFRTGM pBR322 carrying the gentamicin resistance gene and FRT cassette

pKD46 Temperature-sensitive plasmid carrying lambda Red genes ^42^

(*exo*, *bet*, *gam*)

pURS2 *sdeXY* ORF and promoter region cloned into pUC19 This study

pURS3 *sdeAB* ORF and promoter region cloned into pUC19 This study

pURS4 *sdeCDE* ORF and promoter region cloned into pUC19 This study

pURS44 *sdeCD* ORF and promoter region cloned into pUC19 This study

pURS45 *sdeCE* ORF and promoter region cloned into pUC19 This study

pURS5 *sdeGH* ORF and promoter region cloned into pUC19 This study

pURS6 *sdeIJ* ORF and promoter region cloned into pUC19 This study

pURS7 *sdeNO* ORF and promoter region cloned into pUC19 This study

pURS8 *sdePQ*-*omsA* ORF and promoter region cloned into pUC19 This study

pURS82 *sdePQ* ORF and promoter region cloned into pUC19 This study

pURS9 *sdeS* ORF and promoter region cloned into pUC19 This study

pSOS2 *hasF* ORF cloned into pSTV28 This study

pSMXY *sdeXY* ORF and promoter region cloned into pSTV28 This study

**Supplementary Table S3**

**Primers used in the present study**

SMA0370-0369 fw EcoRI CGGAATTCGCACATATAATTAACGCAGCAATGG

SMA0370-0369 re XbaI GTCTAGAGCATTAAGCAACTGAAGCAAAAGAA

SMA0370-0369 re BglII CAGAGATCTGCATTAAGCAACTGAAGCAAAAGAA

SMA1197-1196 fw XbaI ATATCTAGAGGGAAAGCCCGGAAAACAGGG

SMA1197-1196 re EcoRI CGAATTCCGCCTGCTGGTATTTCAAGTCGG

SMA2945-2947F fw EcoRI GGGAATTCACTTATTAAGACAATGCTGACGGTT

SMA2945-2947F re BamHI TTGGATCCGTCCCTTGGCATATTGAGATTTTTTT

SMA2945-2947B fw EcoRI GATCGAATTCTATGGCAAGTGGCTGAAAAC

SMA2945-2947B re BamHI ATCCAGGGATCCCATAAAGA AGCCGAATGC

SMA1059-1060 fw EcoRI CGGAATTCGCTTTGGCTGTAAAATCAGGAAGA

SMA1059-1060 re KpnI AAGGTACCGGCTGCAAAAACTGACGAAAAAAC

SMA1255-1254 fw KpnI TGGGTACCCGACAAATCATTCACCTTATGG

SMA1255-1254 re EcoRI CGGAATTCTTATCAGCGCTTAACCTTTTCG

SMA1698-1699 fw XbaI TTTCTAGATAATTAAGGGAACCCATGCTCGGA

SMA1698-1699 re KpnI CCGGTACCATTTTTTTATCCTTCAGACGTG

SMA1743-1741 fw XbaI CGTCTAGAACTTTTACCGAATCCTTACGCAAC

SMA1743-1741 re EcoRI CCGAATTCGTTTTCTGTTTCTTTACACTTTCCA

SMA2891 fw KpnI CGGGGTACCTCCTTTTTTTCTCCACGCTTT

SMA2891 re EcoRI CGGAATTCACTGAAACACCTTGGGAACGAG

SMA3509 fw EcoRI GGGAATTCGGCAACTATTTCGTCTATCG

SMA3509 re BamHI CCGGATCCAAACGAGAGTGAAGATGATA

sdeXY-FRTfw TTCAGGCAGCTTAGTGCTAACAGGATGTAACGATAAAGAAGCTTTTCTGTGACTGGTGAG

sdeXY-FRTre CACGTTGTCCTTTTCCTTGTTCAGGAAGTAGTCGGAGACTCTATTCTCAGAATGACTTGG

sdeXY-Nfw ATTGAACAAGGATTCATGGC

sdeXY-Nre TTCTTTATCGTTACATCCTG

sdeXY-Cfw AGTCTCCGACTACTTCCTGA

sdeXY-Cre ACCTGGAAGTACACGTCGTT

The recognition site of each restriction enzyme was underlined.
